# Roughness affects the response of human fibroblasts and macrophages to sandblasted abutments

**DOI:** 10.1186/s12938-024-01264-6

**Published:** 2024-07-17

**Authors:** Francisco Romero-Gavilán, Carlos Arias-Mainer, Andreia Cerqueira, David Peñarrocha-Oltra, Juan Carlos Bernabeu-Mira, Iñaki García-Arnáez, Félix Elortza, María Muriach, Mariló Gurruchaga, Isabel Goñi, Julio Suay

**Affiliations:** 1 Department of Industrial Systems Engineering and Design, Universitat Jaume I, Av. Vicent Sos Baynat s/n, 12071 Castellón de la Plana, Spain; 2 Oral Surgery Unit, Department of Stomatology, Faculty of Medicine and Dentistry, University of Valencia, C/Gascó Oliag 1, Valencia, Spain; 3 Departament of Polymers and Advanced Materials: Physics, Chemistry and Technology, Universidad del País Vasco, P. M. de Lardizábal, 3, 20018 San Sebastián, Spain; 4 Proteomics Platform, CIC bioGUNE, Basque Research and Technology Alliance (BRTA), CIBERehd, ProteoRed-ISCIII, Bizkaia Science and Technology Park, 48160 Derio, Spain; 5 Unidad Pre-Departmental de Medicina, Universitat Jaume I, Av. Vicent Sos Baynat s/n, 12071 Castellón de la Plana, Spain

**Keywords:** Soft-tissue healing, Peri-implant attachment, Titanium implants, Proteomics, Protein adsorption

## Abstract

**Background:**

A strong seal of soft-tissue around dental implants is essential to block pathogens from entering the peri-implant interface and prevent infections. Therefore, the integration of soft-tissue poses a challenge in implant-prosthetic procedures, prompting a focus on the interface between peri-implant soft-tissues and the transmucosal component. The aim of this study was to analyse the effects of sandblasted roughness levels on in vitro soft-tissue healing around dental implant abutments. In parallel, proteomic techniques were applied to study the interaction of these surfaces with human serum proteins to evaluate their potential to promote soft-tissue regeneration.

**Results:**

Grade-5 machined titanium discs (MC) underwent sandblasting with alumina particles of two sizes (4 and 8 μm), resulting in two different surface types: MC04 and MC08. Surface morphology and roughness were characterised employing scanning electron microscopy and optical profilometry. Cell adhesion and collagen synthesis, as well as immune responses, were assessed using human gingival fibroblasts (hGF) and macrophages (THP-1), respectively. The profiles of protein adsorption to the surfaces were characterised using proteomics; samples were incubated with human serum, and the adsorbed proteins analysed employing nLC–MS/MS. hGFs exposed to MC04 showed decreased cell area compared to MC, while no differences were found for MC08. hGF collagen synthesis increased after 7 days for MC08. THP-1 macrophages cultured on MC04 and MC08 showed a reduced TNF-α and increased IL-4 secretion. Thus, the sandblasted topography led a reduction in the immune/inflammatory response. One hundred seventy-six distinct proteins adsorbed on the surfaces were identified. Differentially adsorbed proteins were associated with immune response, blood coagulation, angiogenesis, fibrinolysis and tissue regeneration.

**Conclusions:**

Increased roughness through MC08 treatment resulted in increased collagen synthesis in hGF and resulted in a reduction in the surface immune response in human macrophages. These results correlate with the changes in protein adsorption on the surfaces observed through proteomics.

**Supplementary Information:**

The online version contains supplementary material available at 10.1186/s12938-024-01264-6.

## Background

Peri-implantitis is an infectious process observed after implant osseointegration, affecting the hard and soft tissues surrounding the implant [1]. Peri-implantitis develops in 16–28% of patients in the short or long term and may lead to implant loss [2]. Therefore, many researchers have been involved in projects seeking enhancements in implant surface modifications to improve its antimicrobial properties [3]. A considerable effort has been directed towards achieving a robust soft-tissue seal around the implant to prevent the entry of pathogens into the peri-implant interface.

Typically, the oral mucosa barrier protects periodontal tissue against bacteria and other harmful stimuli. Nevertheless, implant placement breaches this barrier, disrupting its continuity [4]. Conventional smooth titanium (Ti) abutments exhibit significantly poorer soft-tissue integration than natural teeth, which jeopardises implant success [5]. Establishing a soft-tissue barrier that shields the underlying peri-implant structures and the implant itself has a pivotal role in preventing bacterial colonisation. It is clear that the conventional abutments should be improved by surface modification to enhance the biomaterial–gingiva fibroblast interactions and promote regenerative functions [6].

The roughness and topography of the abutment are critical factors in implant sealing. Mustafa et al*.* [7] have discovered that differences in the surface roughness of ceramic abutments affect fibroblast spreading and growth. They have reported that polished surfaces with an arithmetic mean height parameter (Sa) of 0.06 µm exhibit significantly stronger cell attachment than the initially rougher surface (Sa = 0.22 µm), albeit with reduced proliferation capacity. Other studies have revealed that micro-grooved Ti surfaces are associated with enhanced fibroblast adhesion and activation compared to polished surfaces [8]. Milled Ti6Al4V (grade 4) surfaces increase gingival fibroblast (hGF) proliferation within the range of 0.3–0.5 µm Sa [9]. Moreover, Cao et al*.* [10] have found that both the adhesive strength and proliferation capability of hGFs were lower on rougher Ti surfaces (Sa = 2.979 µm). To date, there is no firmly established range of roughness for improving gingival sealing nor a consensus on the effect of this parameter. Therefore, further investigation is required.

The process of tissue regeneration around a biomaterial relies on multiple pathways (e.g., complement system, coagulation, fibrinolysis) that affect the level of soft-tissue sealing [11]. When the abutment contacts the bodily fluids, a protein layer forms on its surface; the process is affected by various surface parameters such as roughness [12]. This layer can trigger biological processes that dictate subsequent regenerative responses [13]. Consequently, proteomic techniques have been proven highly valuable for evaluating protein adsorption on biomaterials and studying their interactions with biological tissues [14, 15].

The objective of the present study was to examine the effect of changing roughness on the response of hGFs and THP-1 cells. The Ti surfaces were submitted to different sandblasting treatments, and in vitro assays were conducted. The protein layer was characterised using mass spectrometry techniques.

## Results

### Morphological characterisation

**Fig. 1:**
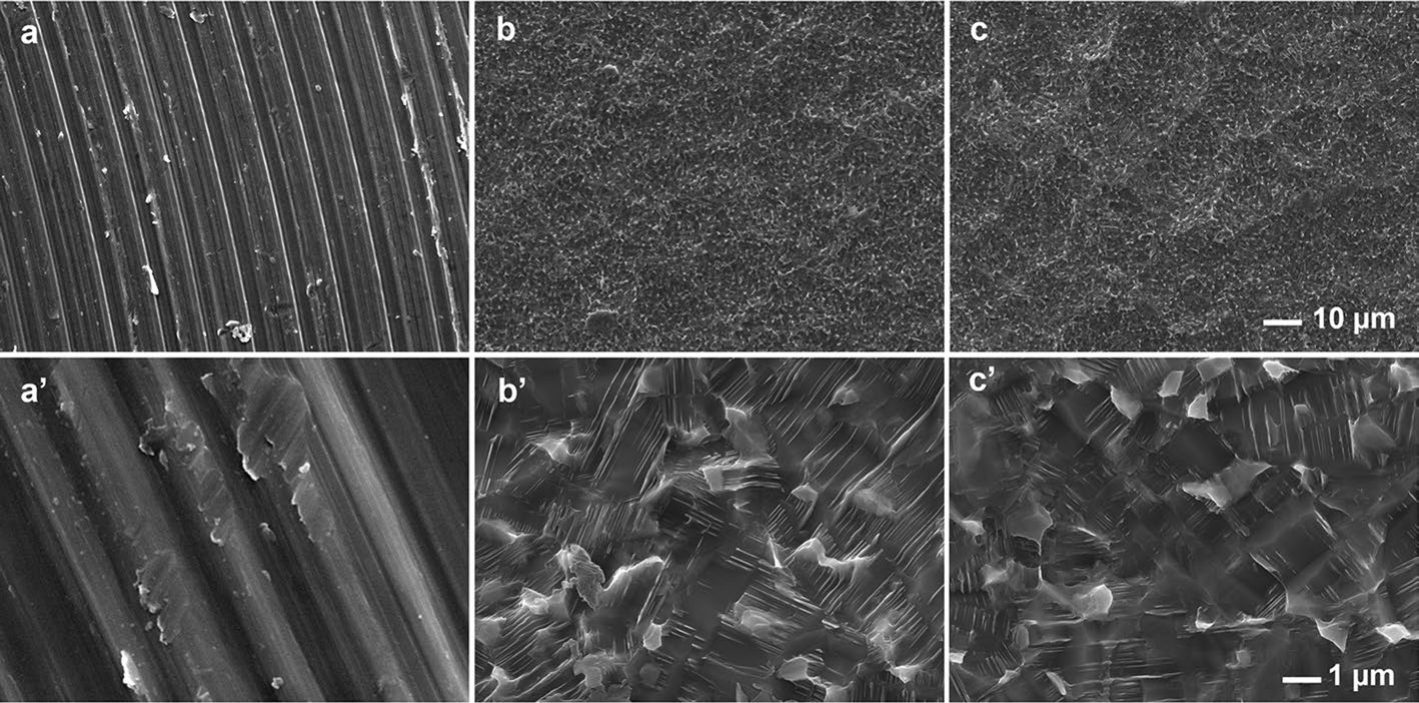
SEM images of MC (**a**, **a′**), MC04 (**b**, **b′**), and MC08 (**c**, **c′**) surfaces. Scale bars, 10 µm (**a**–**c**) and 1 µm (**a′**–**c′**)

The morphology of machined Ti samples (control) and the sandblasted surfaces was analysed by SEM (Fig. 1). In the MC samples (Fig. 1a), marks resulting from the disc machining process can be observed. This initial surface morphology changes completely after sandblasting treatments (Fig. 1b, c). The sandblasted samples exhibit a significant increase in surface irregularities; the treatment leads to the formation of numerous small ridges and valleys. Moreover, these irregularities are more pronounced on the micrometric scale in the MC08 samples due to the larger particle size used in the sandblasting.

**Fig. 2:**
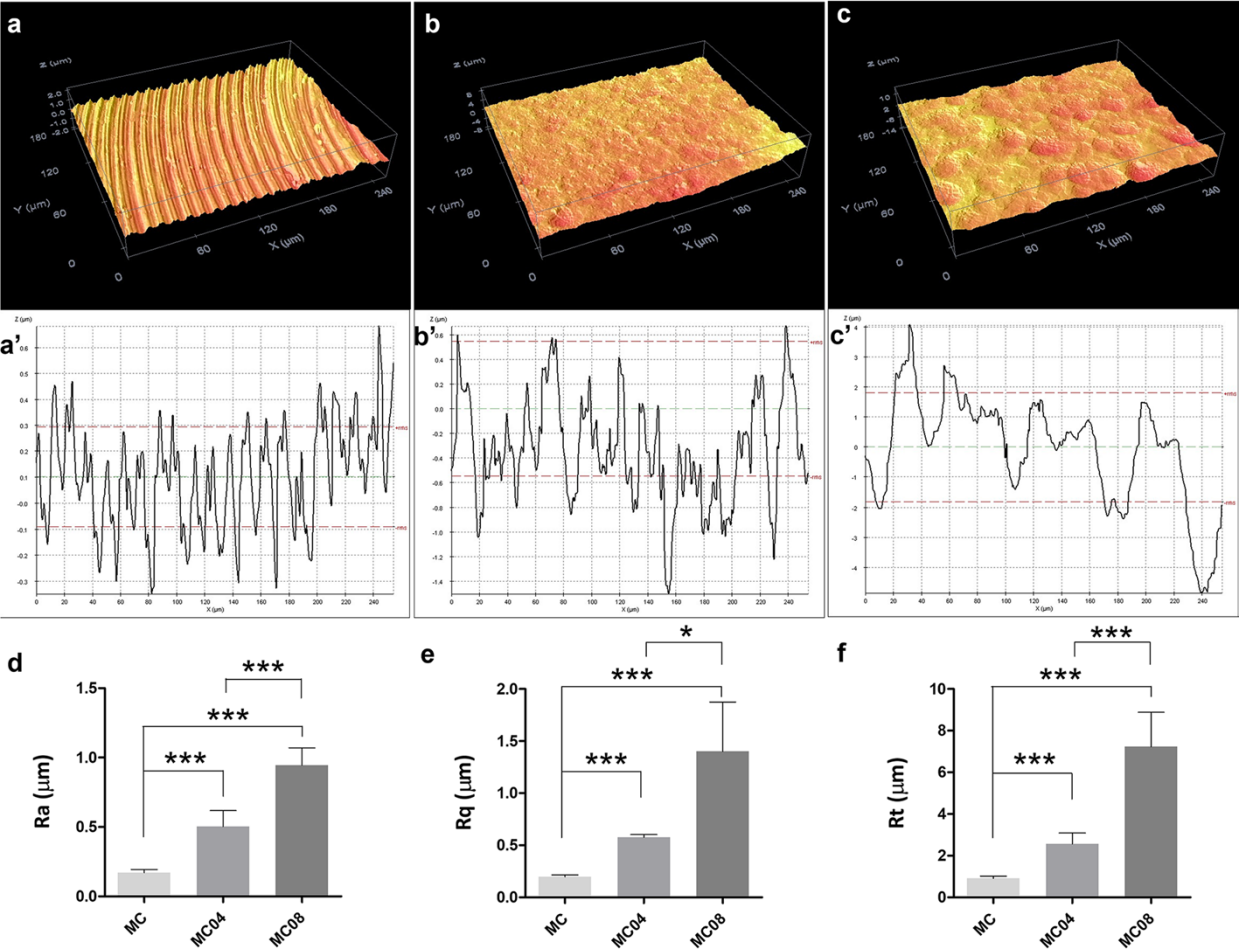
Optical profilometry results for MC (**a**, **a′**), MC04 (b-b′), and MC08 (**c**, **c′**) surfaces. 3D (**a**–**c**) and 2D (**a′**-**c′**) profiles. Arithmetic average of roughness (Ra; **d**), root mean square roughness (Rq; **e**), and total height of the profile (Rt; **f**) measurements. Data are presented as mean ± SD. Statistical analysis was performed using one-way ANOVA with Tukey post hoc test (***p < 0.001)

Consistent with SEM micrographs, the 3D and 2D profiles measured using optical profilometry showed the changes in the surface morphology of the discs due to sandblasting. Indeed, optical profilometer measurements revealed Ra values of approximately 0.17, 0.50 and 0.95 µm for MC, MC04 and MC08, respectively (Fig. 2). This increase in surface roughness could also be evidenced through the increase in the values of Rt and Rq (MC < MC04 < MC08). Thus, the sandblasting process resulted in a significant increase in roughness compared to MC, and MC08 exhibited higher roughness than MC04.

### In vitro assays

#### Cell adhesion and collagen secretion

**Fig. 3:**
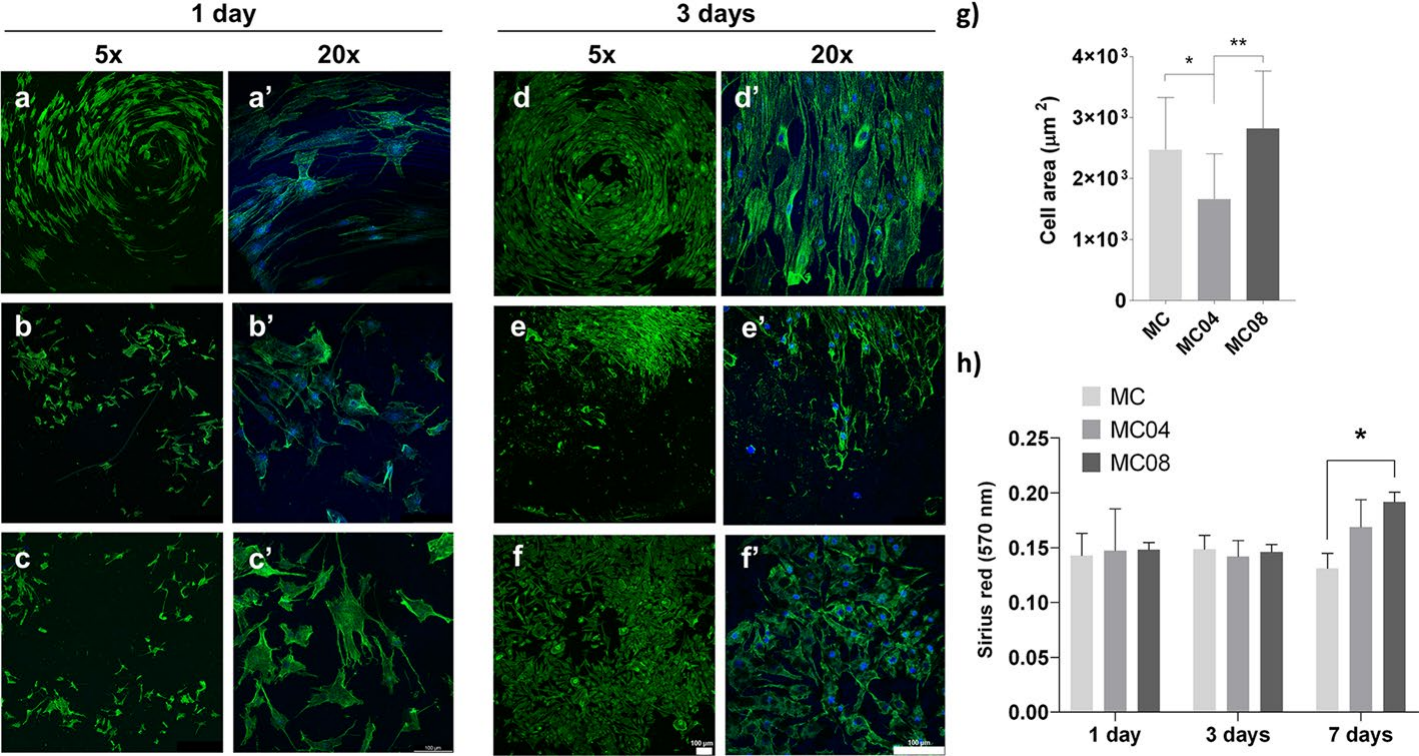
Confocal fluorescence microscopy images of the cytoskeleton arrangement of human gingival fibroblasts (hGF) grown on MC (**a**, **a'** and **d**, **d'**), MC04 (**b**, **b'** and **e**, **e'**) and MC08 (**c**, **c'** and **f**, **f'**) surfaces after 1 and 3 days of culture. Actin filaments were stained with phalloidin (green) and nuclei with DAPI (blue). Scale bar: 100 μm. hGF cell area for the different surfaces after 1 day of culture (**g**). Collagen secretion by hGF on MC, MC04 and MC08 after 1, 3 and 7 days of culture (**h**). Data are presented as mean ± SD. Statistical analysis was performed using one-way ANOVA with the Tukey post hoc test (*p < 0.05; **p < 0.01)

Figure 3a–f shows the cytoskeleton organisation of hGFs cultured on the materials for 1 and 3 days. The cells exhibited good adherence to all the surfaces tested. However, there were differences in growth patterns on MC04 and MC08 compared to MC. While on the MC, fibroblasts tended to adhere and grow in the direction of the circular grooves resulting from the machining process, this effect was not as pronounced on the sandblasted surfaces. After 1 day, the cell area (Fig. 3g) decreased for the MC04 surface compared to MC, while culturing with MC08 did not significantly affect this parameter (compared to MC). The ability of the studied surfaces to promote fibroblast collagen secretion is shown in Fig. 3h. No differences in collagen secretion were observed after 1 and 3 days of culture. However, after 7 days, the cultures with MC08 increased their collagen secretion in comparison with cells incubated with MC.

#### Immune response

**Fig. 4:**
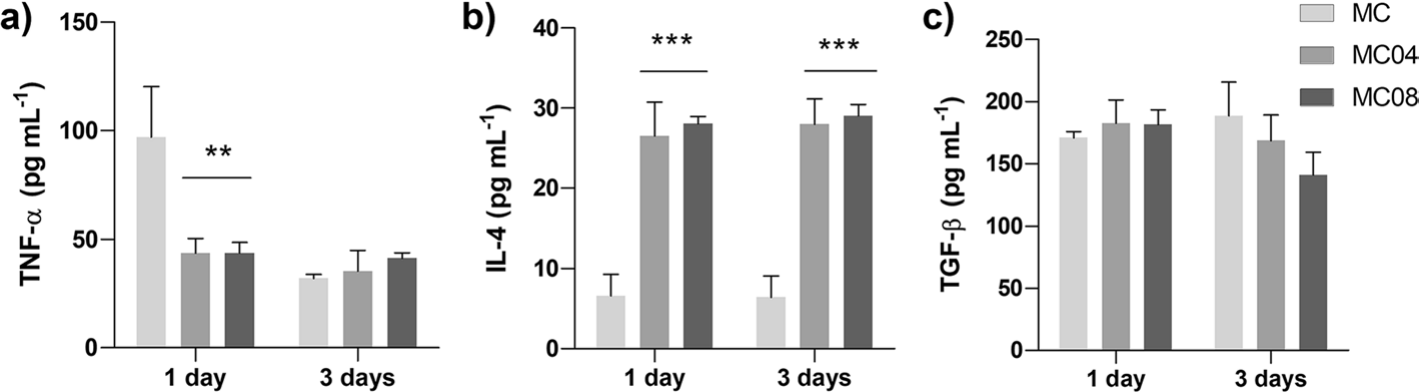
THP-1 cytokine release after 1 and 3 days of culture: **a** tumour necrosis α (TNF-α), **b** interleukin 4 (IL-4), and **c** transforming growth factor beta (TGF-β). Results are shown as mean ± SD. The asterisks (*p* ≤ 0.01 (**) and *p* ≤ 0.001 (***)) indicate statistically significant differences with respect to MC

To assess the impact of surface roughness on the inflammatory response, the secretion of TNF-α, IL-4 and TGF-β cytokines by THP-1 macrophages was measured using ELISA (Fig. 4). After 1 day, a significant decrease in TNF-α secretion by cells cultured on MC04 and MC08 was observed compared to those exposed to MC (Fig. 4a). Moreover, an increase in IL-4 secretion was detected for MC04 and MC08, compared to MC, after 1 and 3 days of culture (Fig. 4b). No differences in TGF-β secretion were found at either of the time points, for any of the materials (Fig. 4c).

### Proteomic analysis

The nLC–MS/MS analysis identified 176 distinct proteins adhered to the surfaces under study. To compare the effects of the sandblasting treatments (MC04 and MC08) with MC, Perseus comparative analyses were conducted (Additional file 1). The results revealed that 24 proteins were differentially more adsorbed onto MC04 compared with MC, while 9 proteins were less adsorbed. MC08 exhibited stronger affinity to 12 proteins than MC and reduced affinity to 8 proteins. Based on the UniProt database, the proteins differentially adsorbed on the evaluated surfaces were categorised according to their functions in immune response, coagulation, angiogenesis and fibrinolysis, and tissue regeneration (Table 1). In general, the adsorption of immunoglobulins and proteins related to the complement system activation (e.g., CO6, CO8A, CO9) increased on the sandblasted surfaces. Moreover, APOL1, related to the innate immune response, showed a higher affinity for these surfaces, and larger amounts of VTNC, a potent inhibitor of the complement system, adsorbed on MC04. In contrast, proteins such as SAMP, DCD, G3P, C1QA, PROP and FHR5, all related to the immune response, showed decreased adsorption on the sandblasted surfaces. Proteins with functions in blood clotting and fibrinolysis, KNG1, KLKB1, PLMN and ANT3, were differentially more adsorbed onto MC04 and MC08. F11 and ZPI proteins were more abundant on MC04. FA5 and HABP2 showed decreased affinity for MC04 and MC08, respectively. Among the proteins associated with tissue regeneration, IBP4 and IGF2 displayed increased adsorption on MC04, but DSC1 and RET4 showed reduced affinity to this surface. Moreover, smaller amounts of DSC1 and DSG1 adsorbed onto MC08 than to MC.

**Table 1:** Functional analysis of proteins differentially adsorbed onto MC04 and MC08 compared to MC. The proteins were categorised into three functional groups: immune response, coagulation and fibrinolysis, and tissue regeneration

		MC04/MC	MC08/MC
Immune response	UP	HV169, CO6, CO9, CO8A, HV372, VTNC, KV401, HV551, HV459, CO5, CRP, APOL1	HV313, CO6, CO9, CO8A, HV372, APOL1
	DOWN	C1QA, SAMP, G3P, DCD	SAMP, FHR5, G3P, PROP, DCD
Coagulation and fibrinolysis	UP	KNG1, KLKB1, FA11, PLMN, ANT3, ZPI	KNG1, KLKB1, PLMN, ANT3
	DOWN	FA5	HABP2
Tissue regeneration	UP	IBP4, IGF2	–
	DOWN	DSC1, RET4	DSC1, DSG1

**Fig. 5:**
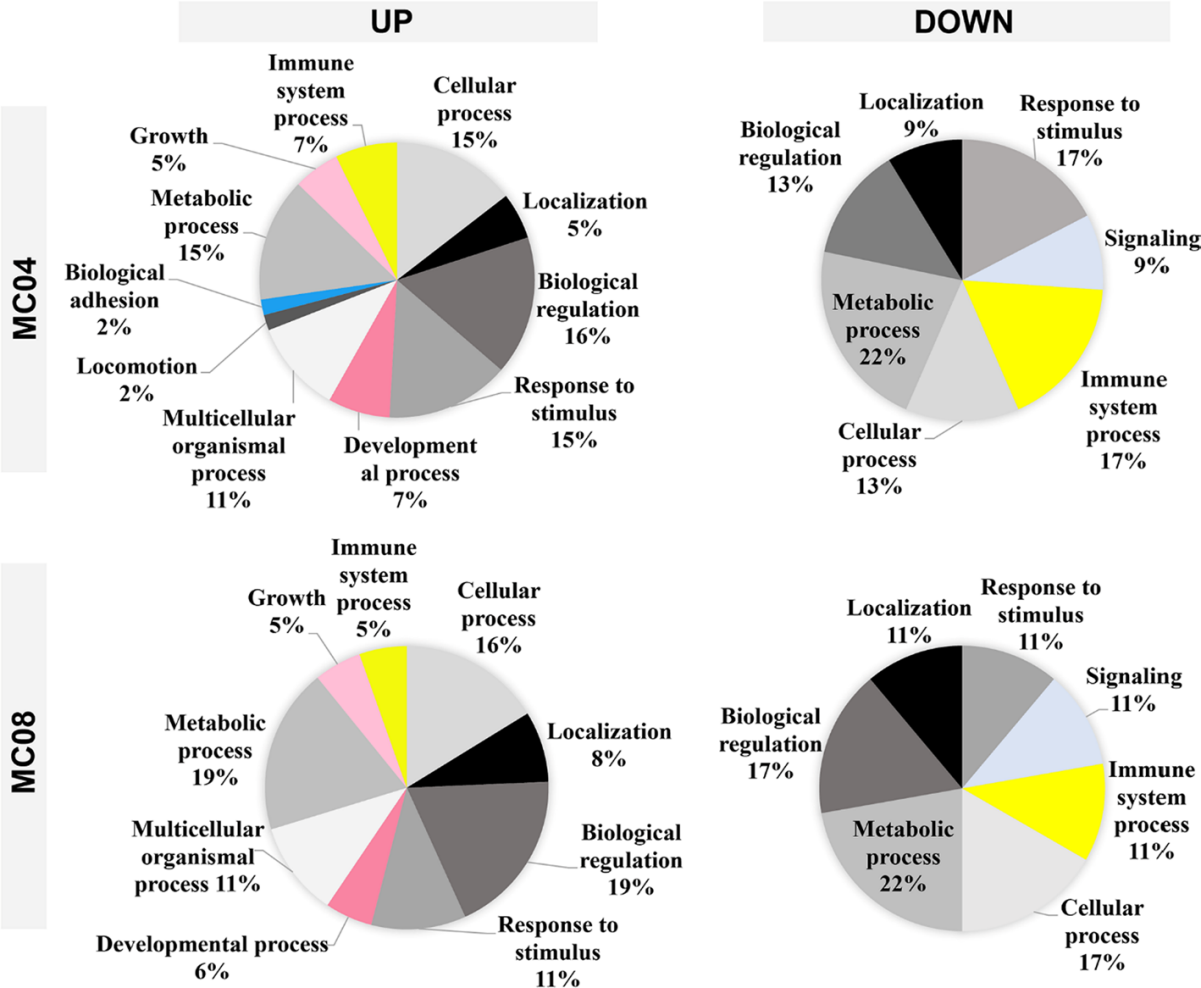
Pie charts depicting the PANTHER classification of biological functions associated with the UP and DOWN differential proteins for MC04 and MC08

Furthermore, a functional analysis was conducted using PANTHER and STRING. In PANTHER analysis (Fig. 5), the overall classification obtained for proteins showing increased affinity on MC04 was similar to that for the proteins most adsorbed on MC08. The enhanced functions were linked to biological regulation, growth, development, metabolic, cellular or immune system processes. In contrast, proteins with reduced adsorption on the MC04 and MC08 materials generated profile charts with biological functions primarily associated with the metabolic process, response to stimuli or the immune system.

**Fig. 6:**
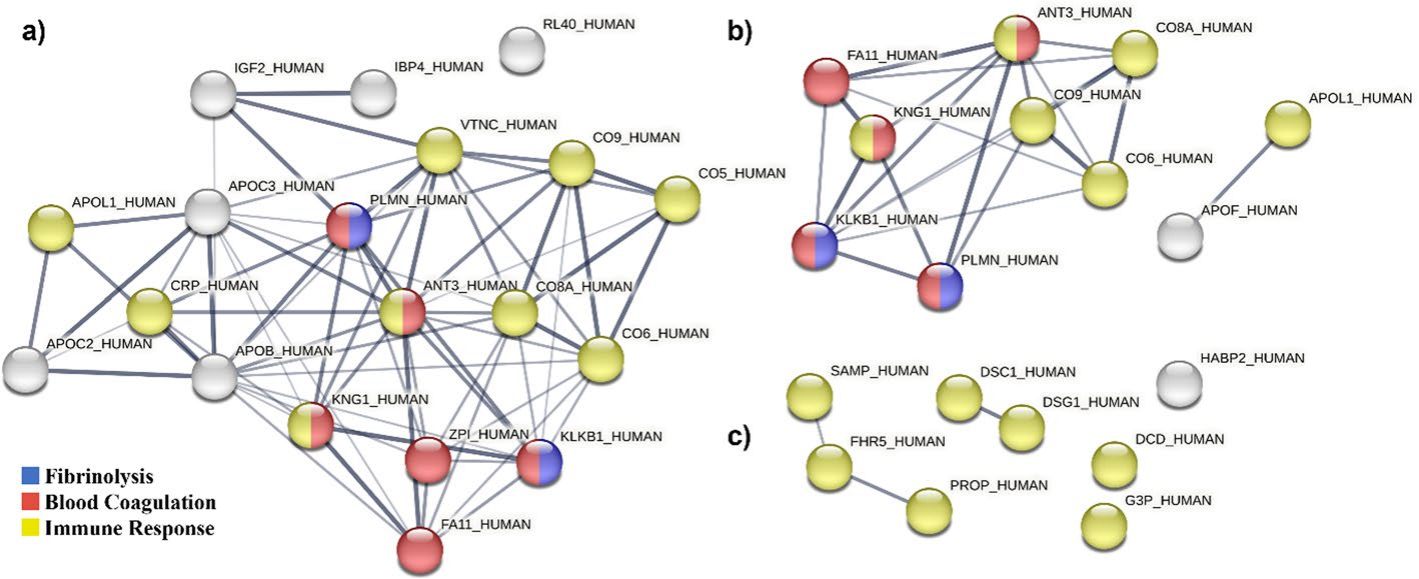
STRING interaction networks between the differentially adsorbed proteins with stronger affinity to MC04 (**a**) and MC08 (**b**) and weaker affinity to MC08 (**c**) than to MC. Line thickness indicates the interaction strength. The colours represent enriched functions associated with the protein network.

The interaction networks formed by the proteins differentially adsorbed on MC04 and MC08 are presented in Fig. 6. For proteins with increased affinity for MC04, a protein network enriched in blood coagulation, fibrinolysis, and immune functions was identified (Fig. 6a).

Proteins differentially adsorbed on MC08 (compared to MC) were associated with four networks. Those with greater affinity for MC08 formed one network with proteins linked to blood coagulation, fibrinolysis, and the immune response, and another network with two apolipoproteins related to immune functions (Fig. 6b). Proteins with reduced affinity to MC08 formed two interaction networks with an enrichment in the immune response (Fig. 6b').

## Discussion

The regeneration of peri-implant soft tissue is governed by a complex interplay of growth factors, cytokines and matrices, orchestrating a series of interconnected phases: inflammation, new tissue generation and matrix remodelling [4]. The topography of the abutment can affect these processes and, consequently, the extent of gingival sealing achieved around the implant. However, while much research has focused on the effect of dental implant topography on osseointegration [18, 19], there is no consensus on its impact on the prosthesis in contact with soft tissue. This study explored the relationship between the surface roughness of grade 5-Ti abutments and their in vitro behaviour in terms of cell adhesion, collagen secretion and immune response. Moreover, the observed biological responses were correlated with the adsorption of human serum proteins.

The two sandblasting treatments (MC04 and MC08) resulted in a significant increase in roughness, from 0.17 µm Ra on MC to 0.50 and 0.95 µm on MC04 and MC08, respectively. These morphological changes led to alterations in the protein adsorption patterns on the evaluated surfaces. The proteomic analysis revealed that 33 proteins adsorbed differentially onto MC04 and 20 onto MC08. Changes in protein adsorption to the abutment surfaces can be affected by sandblasting; the biomaterial surface roughness has been identified as a key factor in this phenomenon [20]. These differentially adsorbed proteins have functions related to coagulation, fibrinolysis, immunity and tissue regeneration, which may impact the subsequent biological responses [21].

The study of the response of human gingival fibroblasts (hGFs) to the examined surfaces showed a reduction in cell area in cultures with MC04 compared to MC and MC08. Interestingly, a study by Akiyama et al*.* [22] evaluated the effect of grade 4 machined Ti with different levels of roughness (0.98 and 0.02 µm, Ra) on hGFs. They found no differences in proliferation, adhesion, and collagen synthesis of these cells due to the Ra variation. In another work, polishing treatments of grade 2 Ti resulted in distinct Ra values ranging from 0.075 to 1.024 µm. In this case, the authors did not observe significant differences in hGF attachment [23]. Otherwise, in another study, the hGFs cultured on rough sandblasted acid-etched Ti (SLA) were compared to those cultured on smooth machined Ti [24]. Noticeably different fibroblast morphologies were observed on the two surfaces. The cells on machined samples had an elongated morphology and were attached along the grooves, whereas they were randomly distributed on the SLA. Moreover, the hGFs showed increased expression of focal adhesion kinase (FAK), α-smooth muscle actin (α-SMA) and integrin subunits ITG-β4, ITG-α5 and ITG-α6 on the rough surfaces. The results of that study are consistent with the distinct fibroblast adhesion patterns observed for MC04 and MC08 compared to MC. Wieland et al*.* [25] studied different topographies obtained by surface modifications typically applied to Ti prostheses and found that surface features affected hGF thickness and morphology, but not to its volume. These results suggest that cells adapt their shape to attach to rough surfaces depending on the material morphology. The capability of cells to adjust their shape while binding to different surfaces could explain the reduction in cell area observed for MC04. However, this smaller cell area is no longer detected with increasing Ra for M08. Additionally, cells cultured on MC08 increased collagen secretion in comparison with MC. It appears that the fibroblast response to these surfaces depends not only on roughness, but also on the morphology resulting from the surface treatment. On the other hand, the possible influence of roughness on the formation of bacterial biofilm causes some authors to establish that machined or polished surfaces should be preferred over rougher surfaces for dental abutments [26]. However, the increased roughness due to sandblasting does not necessarily promote bacterial proliferation [27]. In fact, Petrini et al*.* [28] evaluated the capability of *Streptococcus oralis* to adhere to a double-etched titanium (DAE), in respect to machined and single-etched titanium. Despite the increased roughness of DAE surface, this treatment displayed the best performance in terms of inhibiting bacterial adhesion. It seems that the key in this sense is in the scale at which the roughness is measured (micro- and nano-roughness). While bacteria demonstrated insensitivity to micrometric irregularities [27], their adhesion could be conditioned by surface nano-roughness [29].

The current study showed that sandblasting affected the immune response. Compared to MC cultures, the THP-1 cells decreased TNF-α and increased IL-4 secretion when grown on MC04 and MC08. Macrophages play a pivotal role in tissue regeneration and inflammation; their M1 phenotype produces proinflammatory cytokines like TNF-α [30]. In contrast, IL-4 is considered a marker for macrophage polarisation towards the pro-healing M2 phenotype [31]. Our results suggest that sandblasted dental abutments may have an immunomodulatory effect by promoting macrophage polarisation towards the M2 phenotype. This phenomenon can be correlated with the changes in the adsorption, on MC04 and MC08, of proteins associated with immune responses. RAW 264.7 macrophages seeded on rough SLA surface displayed higher IL-6 and TNF-α gene expression in comparison to pickled Ti. This increased proinflammatory reaction on SLA was related to a negative impact on gingival fibroblast behaviour on titanium surfaces [32]. Similarly, RAW264.7 macrophages cultured on nanostructured zirconia surfaces showed increased TNF-α production for the surface with higher nano-roughness, whereas the smoother treatment provoked a higher IL-10 secretion [33]. However, while smooth, micro-rough, and nano-rough Ti surfaces did not affect THP-1 macrophage polarisation, the micro- and nano-rough surfaces demonstrated improved attachment of human gingival fibroblasts compared to the smooth samples [34]. Consistent with our results, Lackington et al*.* [35] found that rough surfaces stimulated the THP-1 macrophage activation towards an anti-inflammatory (M2-like) phenotype; with decreased secretion of TNF-α, IL-6 and IL-8 in the rough surfaces compared to Ti machined treatment.

Proteins associated with the activation of the complement cascade (CRP, CO5, CO6, CO9, CO8A) and immunoglobulins (HV169, HV372, KV401, HV551, HV459, HV313) showed increased affinity to the rough surfaces. The complement system plays an integral role in innate immunity by triggering local inflammation and clearance mechanisms [36]. However, the VTNC protein, which plays a key role in regulating the complement system activation [37], also increased its adsorption. Moreover, C1QA, a complement system protein, and the pentraxin SAMP, related to immune response activation [38], showed decreased affinity to sandblasted surfaces. It is also noteworthy that there was a significant decrease in the adsorption of DCD and G3P proteins on the rough surfaces compared with MC. G3P, also known as GAPDH, is essential for cytokine production in macrophages and can regulate TNF-α production through RNA binding [39]. DCD actively participates in the constitutive innate immune defence of human skin against infection [40]. Its isoform-2 induces the expression of TNF-α and IL-6 inflammatory cytokines via nitric oxide synthesis in human neutrophils [41]. The PROP protein, also less adsorbed to MC08 than to MC, is an upregulator of the complement activation pathway. This protein can enhance the macrophage proinflammatory response, resulting in higher production of TNF-α, IL-1β and IL-1α proinflammatory cytokines while suppressing the anti-inflammatory cytokines IL-10 and TGF-β [42]. The effect of sandblasting on the adsorption of proteins associated with immune functions is also evident in the classifications obtained using PANTHER. Approximately 5–7% of the proteins identified as upregulated on MC04 and MC08 were associated with the immune system process, and 17% of the downregulated proteins were related to this biological function. Thus, the surface modifications applied here resulted in a complex balance between increased adsorption of proinflammatory proteins and a substantial reduction of other proteins with similar functions, ultimately creating an anti-inflammatory environment in vitro.

Insulin growth factors, such as IGF1 and IGF2, can be associated with fibroblast proliferation [43]. In the current study, IGF2 and IBP4 were more abundant on MC04 than on MC. IBP4 is known to inhibit IGF-1 activity [44], which can activate TNF-α production in monocytes [45]. Du et al*.* [46] have found that IGF2 modulates the innate immune memory of macrophages during their maturation. This maintains persistent oxidative phosphorylation, which determines their anti-inflammatory phenotype. RET4, a downregulated protein in MC04, can inhibit insulin signalling by inducing proinflammatory cytokines in macrophages [47]. The adsorption patterns of IGF2, IBP4 and RET4 correlated with the anti-inflammatory potential displayed in vitro.

Furthermore, proteomic results revealed differential adsorption to sandblasted surfaces of proteins related to coagulation and fibrinolysis. Proper blood clotting is essential to form the fibrin matrix that supports tissue regeneration [4]. Williams et al*.* [48] have found that rough surfaces stimulate the formation of denser fibrin networks than smooth ones, favouring the integration of soft tissue. The KNG1 and KLKB1proteins, preferentially more adsorbed on MC04 and MC08 materials, activate the kallikrein–kinin system, a pathway capable of initiating blood coagulation [49]. Similarly, the coagulation factor FA11, with increased affinity for MC04, can activate the blood coagulation cascade to facilitate the formation of cross-linked fibrin clots [49]. However, FA5, another factor in this cascade [50], was less abundant on that surface. Increased adsorption of ANT3 and ZPI serpin inhibitors to sandblasted surfaces was also detected; these proteins are associated with anticoagulant functions [49]. Following the formation of a blood clot, the fibrinolysis process is crucial for wound healing. PLMN, found to be more adsorbed on MC04 and MC08 than on MC, plays a pivotal role in this biological process, enabling the degradation and remodelling of the fibrin matrix [51]. Plasminogen deficiency disorder has been associated with aggressive periodontitis and gingival enlargement [52].

The protein adsorption profiles and the cellular responses to sandblasted Ti samples indicate the potential of these surfaces, especially MC08, in the modification of dental abutments. This surface could be optimal at a morphological level to reach a correct soft tissue regeneration. However, in the future, the study of the effect of this surface on the bacterial biofilm formation and its regeneration capacity in vivo will confirm the ability of the MC08 to achieve effective soft-tissue sealing around abutments.

## Conclusion

The degree of surface roughness affects cellular responses; this effect also depends on the morphological changes in the treated surfaces. The sandblasted samples, MC04 and MC08, exhibited significant surface irregularities, characterised by numerous small ridges and valleys, compared to the smoother MC surface. This resulted in increased roughness, with Ra values of 0.50 µm for MC04 and 0.95 µm for MC08. The cytoskeleton organisation of human gingival fibroblasts (hGFs) on MC04 was reduced compared to MC, likely due to the combined effect of surface roughness and changed morphology. In contrast, culturing with MC08 (with an Ra of 0.95 µm) resulted in cells with enlarged areas (in comparison with MC04) and higher collagen secretion levels than the cells exposed to machined Ti or MC04. Moreover, surface modification by sandblasting had an impact on the immune response of the macrophages. Culturing with MC04 or MC08 led to an increase in IL-4 release and a decrease in TNF-α concentration, suggesting an anti-inflammatory response. Proteomic analysis revealed that the proteins with higher affinity for MC04 and MC08 were mainly associated with coagulation, fibrinolysis, immunity and tissue regeneration, which might explain the observed in vitro results.

## Methods

### Titanium surface preparation

Grade-5 titanium discs (6-mm diameter; Nueva Galimplant S.L., Lugo, Spain) were machined and treated by sandblasting to achieve different levels of roughness. The samples were abraded with aluminium oxide particles of different sizes, 4 μm and 8 μm, resulting in MC04 and MC08 surfaces, respectively. The discs were loaded into the sample holder, which was based on a rotating carousel with six sample holder satellites. Inside the sandblasting cabin, there were three blasting stations: two stations with a single nozzle each, and a central station with two fixed nozzles. All nozzles were positioned 8 cm from the sample, each oriented to cover different sample sections to ensure uniform vertical blasting. At each sandblasting station, the sample was blasted for 3.6 s, with pressure gradually decreasing from 7.4 bar to 4 bar. During this process, the sample holder rotated to ensure uniform blasting around the entire perimeter of the sample. Discs were then cleaned with acetone, ethanol, and Milli-Q water (20 min for each wash) in an ultrasonic bath. Non-sandblasted machined samples (MC) were used as references. The discs were sterilised using UV radiation before use.

### Surface morphological characterisation

A Leica-Zeiss LEO scanning electron microscope (SEM; Leica, Wetzlar, Germany) was employed to assess the surface morphology of the discs before and after sandblasting. To enhance sample conductivity, discs were sputtered with platinum before SEM examination. An optical profilometer PLm2300 (Sensofar, Barcelona, Spain) was used to measure roughness. Three discs were analysed for each treatment, with three measurements taken for each disc. The roughness measurements were reported as the arithmetic average roughness (Ra), the root mean square roughness (Rq), and the total height of the profile (Rt) parameters.

### In vitro testing

#### Cell cultures

The human gingival fibroblasts cell line (hGF; LGC Standards, Barcelona, Spain) was used to assess the behaviour of cells in contact with different surfaces. The hGF cells were employed to examine the cell adhesion and the capacity to promote collagen synthesis. The fibroblasts were cultured in Fibroblast Basal Medium supplemented with 5 ng mL^−1^ recombinant human fibroblast growth factor-basic (rh FGF b), 7.5 mM L­glutamine, 50 µg mL^−1^ ascorbic acid, 1 µg mL^−1^ hydrocortisone hemisuccinate, 5 µg mL^−1^ rh insulin, 2% FBS and 1% penicillin/streptomycin. All reagents were obtained from the American Type Culture Collection (ATCC; Manassas, VA, USA). Human monocytes (THP-1; ECACC, Public Health England, Porton Down, Salisbury, UK) were used to evaluate the immune response to the modified surfaces. The cells were cultured in RPMI-1640 medium supplemented with 10% FBS and 1% penicillin/streptomycin (Merck, Waltham, MA, USA). Phorbol 12-myristate 13-acetate (50 ng mL^−1^; PMA; Merck) was employed to differentiate the THP-1 into macrophages. In both cases, wells without biomaterial samples and with MC discs were used as controls. The cells were maintained under a humidified atmosphere (90% H_2_O) of 5% CO_2_ at 37 °C.

#### Cell adhesion

The hGF cytoskeleton arrangement on the surfaces was evaluated to measure their potential to promote cell adhesion. Fibroblasts were seeded on the discs at a density of 10 × 10^3^ cells cm^−2^ in 96-well plates (Corning Inc.; Somerville, MA, USA) for 1 and 3 days; then, the assay was carried out as previously described [16]. Briefly, cells were washed with PBS and fixed with 4% paraformaldehyde (Thermo Fisher Scientific, Waltham, MA, USA). Then, the samples were permeabilised with 0.1% Triton X-100 and incubated with phalloidin (1:100; Abcam, Cambridge, UK) diluted in 0.1% w/v bovine serum albumin (BSA)-PBS for 1 h. Cell nuclei were stained in a mounting medium with DAPI (Abcam) for 5 min. Fluorescence was measured using a Leica TCS SP8 Confocal Laser Scanning Microscope (Leica), and photographs were taken using LAS X software (Leica). The images were analysed employing the Image J software (National Institutes of Health, Maryland, USA). The assay was performed in triplicate, and 30 photographs were taken for each condition to determine the cell area.

#### Collagen synthesis

The effect of roughness on fibroblast collagen synthesis was evaluated by Sirius Red staining in saturated picric acid (SR; Merck). The hGF cells were seeded on the different biomaterial samples at densities of 20 × 10^3^, 10 × 10^3^, and 5 × 10^3^ cells cm^−2^ in 96-well plates for 1, 3 and 7 days, respectively. Cells were fixed using 4% paraformaldehyde (Thermo Fisher Scientific) for 20 min and washed with PBS. Then, the samples were incubated with the SR solution for 24 h. After washing off the dye residue with water, the stain was extracted from samples using 1 M NaOH (Merck). A Multiskan FC microplate reader (Thermo Fisher Scientific) was used to measure the absorbance at 570 nm. The experiment was carried out in triplicate.

#### Immune response

THP-1 cells were used to assess the effect of roughness on the immune response. The cells were seeded on the sample discs at densities of 3 × 10^5^ and 1.5 × 10^4^ cells cm^−2^ in 96-well plates for 1 day and 3 days, respectively. Then, the culture media were collected, and the tumour necrosis factor α (TNF-α), transforming growth factor β (TGF- β), and interleukin (IL-4) release levels were evaluated using ELISA kits (Invitrogen, Thermo Fisher Scientific) following the manufacturer's guidelines. The assay was performed in triplicate.

### Protein layer characterisation

#### Protein layer formation assay

The effect of roughness on the protein adsorption patterns on the abutment surfaces was analysed using proteomics. The discs were incubated in 96-well plates with 0.2 mL of human serum (from male AB plasma; Merck) under a humidified atmosphere of 5% CO_2_ at 37 °C for 3 h. After this incubation, the samples were washed with ddH_2_O and 100 mM NaCl 50 mM Tris–HCl (pH 7.0; Merck) buffer to remove the non-adsorbed proteins. The proteins adsorbed on the surfaces were eluted using a solution of 2 M thiourea, 7 M urea, 4% CHAPS and 200 mM dithiothreitol (Merck). The experiment was performed in quadruplicate; each replicate was obtained by pooling the eluates from four samples.

#### nLC–MS/MS analysis and bioinformatic data processing

An Evosep ONE chromatograph (Evosep, Odense C, Denmark) was coupled with a hybrid trapped ion mobility-quadrupole time-of-flight mass spectrometer (timsTOF Pro with PASEF; Bruker, Billerica, MA, USA) to analyse the eluted protein layers, as previously described [17]. Each condition was analysed in quadruplicate. Protein identification and quantification were performed using MaxQuant software (http://maxquant.org/), and differential analyses between conditions were carried out using Perseus (https://www.maxquant.org/perseus/). UniProt database (https://www.uniprot.org/) and PANTHER system (http://www.pantherdb.org/) were utilised to categorise differential proteins based on their functions. STRING v.11.5 (Search Tool for the Retrieval of Interacting Genes/Proteins; https://string-db.org/) was employed to assess the interaction networks of differential proteins and identify enriched functions related to these proteins. The UniProt ID codes were used as protein names**.**

### Statistical analysis

One-way variance analysis (ANOVA) with a Tukey post hoc test was applied to identify statistically significant differences (GraphPad Prism® software version 5.04; GraphPad Software Inc., La Jolla, CA, USA) between the physicochemical and in vitro results. Values of *p* < 0.05 were considered statistically significant. For proteomics, the Student's *t*-test in the Perseus software was used to obtain statistical differences in protein adsorption for different surface treatments. To classify a protein as differentially adsorbed, its abundance between conditions had to exhibit statistically significant differences (*p* ≤ 0.05), and its ratio had to be higher than 1.5 in either direction (UP or DOWN).

### Supplementary Information

Additional file 1.

## Data Availability

All data generated or analysed during this study are included in this published article [and its supplementary information files].

## References

[1] Jordana F, Susbielles L, Colat-Parros J (2018). Periimplantitis and implant body roughness: a systematic review of literature. Implant Dent.

[2] Rinke S, Ohl S, Ziebolz D, Lange K, Eickholz P (2011). Prevalence of periimplant disease in partially edentulous patients: a practice-based cross-sectional study. Clin Oral Implants Res.

[3] Chen Z, Wang Z, Qiu W, Fang F (2021). Overview of antibacterial strategies of dental implant materials for the prevention of peri-implantitis. Bioconjug Chem.

[4] Guo T, Gulati K, Arora H, Han P, Fournier B, Ivanovski S (2021). Orchestrating soft tissue integration at the transmucosal region of titanium implants. Acta Biomater.

[5] Atsuta I, Ayukawa Y, Kondo R, Oshiro W, Matsuura Y, Furuhashi A (2016). Soft tissue sealing around dental implants based on histological interpretation. J Prosthodont Res.

[6] Guo T, Gulati K, Arora H, Han P, Fournier B, Ivanovski S (2021). Race to invade: understanding soft tissue integration at the transmucosal region of titanium dental implants. Dent Mater.

[7] Mustafa K, Odén A, Wennerberg A, Hultenby K, Arvidson K (2005). The influence of surface topography of ceramic abutments on the attachment and proliferation of human oral fibroblasts. Biomaterials.

[8] Guillem-Marti J, Delgado L, Godoy-Gallardo M, Pegueroles M, Herrero M, Gil FJ (2013). Fibroblast adhesion and activation onto micro-machined titanium surfaces. Clin Oral Implants Res.

[9] Osman MA, Alamoush RA, Kushnerev E, Seymour KG, Watts DC, Yates JM (2022). Biological response of epithelial and connective tissue cells to titanium surfaces with different ranges of roughness: an in-vitro study. Dent Mater.

[10] Jie C, Wang T, Yinfei P, Zhihui T, Huanxin M (2018). Influence on proliferation and adhesion of human gingival fibroblasts from different titanium surface decontamination treatments: an in vitro study. Arch Oral Biol.

[11] Lyu Z, Yu Q, Chen H. Interactions of biomaterials surfaces with proteins and cells. In: Gao C, editor. Polym biomater tissue regen from surface/interface des to 3D Constr. Singapore: Springer; 2016. p. 103–22. 10.1007/978-981-10-2293-7

[12] Romero-Gavilán F, Gomes NC, Ródenas J, Sánchez A, Mikel A, IbonIloro F, Elortza IGA (2017). Proteome analysis of human serum proteins adsorbed onto different titanium surfaces used in dental implants. Biofouling.

[13] Romero-Gavilán F, Sanchez-Pérez AM, Araújo-Gomes N, Azkargorta M, Iloro I, Elortza F (2017). Proteomic analysis of silica hybrid sol-gel coatings: a potential tool for predicting the biocompatibility of implants in vivo. Biofouling.

[14] Calciolari E, Donos N (2018). The use of omics profiling to improve outcomes of bone regeneration and osseointegration How far are we from personalized medicine in dentistry?. J Proteomics.

[15] García-Arnáez I, Romero-Gavilán F, Cerqueira A, Elortza F, Azkargorta M, Muñoz F (2022). Correlation between biological responses in vitro and in vivo to Ca-doped sol-gel coatings assessed using proteomic analysis. Colloids Surfaces B Biointerfaces..

[16] Cerqueira A, Romero-Gavilán F, García-Arnáez I, Martinez-Ramos C, Ozturan S, Izquierdo R (2021). Characterization of magnesium doped sol-gel biomaterial for bone tissue regeneration: the effect of Mg ion in protein adsorption. Mater Sci Eng C.

[17] Romero-Gavilán F, Cerqueira A, García-Arnáez I, Azkargorta M, Elortza F, Gurruchaga M (2023). Proteomic evaluation of human osteoblast responses to titanium implants over time. J Biomed Mater Res Part A.

[18] Matos GRM (2021). Surface roughness of dental implant and osseointegration. J Maxillofac Oral Surg.

[19] Shirazi S, Ravindran S, Cooper LF (2022). Topography-mediated immunomodulation in osseointegration. Ally Enemy Biomater.

[20] Barberi J, Spriano S (2021). Titanium and protein adsorption: an overview of mechanisms and effects of surface features. Materials (Basel)..

[21] Araújo-Gomes N, Romero-Gavilán F, Sánchez-Pérez AM, Gurruchaga M, Azkargorta M, Elortza F (2018). Characterization of serum proteins attached to distinct sol–gel hybrid surfaces. J Biomed Mater Res Part B Appl Biomater.

[22] Akiyama Y, Iwasa F, Hotta Y, Matsumoto T, Oshima Y, Baba K (2021). Effects of surface roughness of ceria-stabilized zirconia/alumina nanocomposite on the morphology and function of human gingival fibroblasts. Dent Mater J.

[23] Yanagisawa N, Ikeda T, Takatsu M, Urata K, Nishio K, Tanaka H (2022). Human gingival fibroblast attachment to smooth titanium disks with different surface roughnesses. Biomimetics.

[24] Rausch MA, Shokoohi-tabrizi H, Wehner C, Pippenger BE, Wagner RS, Ulm C (2021). Impact of implant surface material and microscale roughness on the initial attachment and proliferation of primary human gingival fibroblasts. Biology (Basel).

[25] Wieland M, Chehroudi B, Textor M, Brunette DM (2002). Use of Ti-coated replicas to investigate the effects on fibroblast shape of surfaces with varying roughness and constant chemical composition. J Biomed Mater Res.

[26] Laleman I, Lambert F (2023). Implant connection and abutment selection as a predisposing and/or precipitating factor for peri-implant diseases: a review. Clin Implant Dent Relat Res.

[27] Rodriguez-González R, Monsalve-Guil L, Jimenez-Guerra A, Velasco-Ortega E, Moreno-Muñoz J, Nuñez-Marquez E (2023). Relevant aspects of titanium topography for osteoblastic adhesion and inhibition of bacterial colonization. Materials (Basel).

[28] Petrini M, Giuliani A, Di Campli E, Di Lodovico S, Iezzi G, Piattelli A (2020). The bacterial anti-adhesive activity of double-etched titanium (Dae) as a dental implant surface. Int J Mol Sci.

[29] Xing R, Lyngstadaas SP, Ellingsen JE, Taxt-Lamolle S, Haugen HJ (2015). The influence of surface nanoroughness, texture and chemistry of TiZr implant abutment on oral biofilm accumulation. Clin Oral Implants Res.

[30] Mamilos A, Winter L, Schmitt VH, Barsch F, Grevenstein D, Wagner W (2023). Macrophages: from simple phagocyte to an integrative regulatory cell for inflammation and tissue regeneration—a review of the literature. Cells.

[31] Spiller KL, Nassiri S, Witherel CE, Anfang RR, Ng J, Nakazawa KR (2015). Sequential delivery of immunomodulatory cytokines to facilitate the M1-to-M2 transition of macrophages and enhance vascularization of bone scaffolds. Biomaterials.

[32] Wang X, Wang Y, Bosshardt DD, Miron RJ, Zhang Y (2018). The role of macrophage polarization on fibroblast behavior-an in vitro investigation on titanium surfaces. Clin Oral Investig.

[33] Wu J, Yu P, Lv H, Yang S, Wu Z (2021). Nanostructured zirconia surfaces regulate human gingival fibroblasts behavior through differential modulation of macrophage polarization. Front Bioeng Biotechnol.

[34] Miao X, Wang D, Xu L. The response of human osteoblasts, epithelial cells, fibroblasts, macrophages and oral bacteria to nanostructured titanium surfaces: a systematic study (Int J Nanomedicine, (2017) 12, (1415–1430), 10.2147/IJN.S126760). Int J Nanomed. 2020;15:2351–2. 10.2147/IJN.S126760 PMC5325133 28260888

[35] Lackington WA, Fleyshman L, Schweizer P, Elbs-Glatz Y, Guimond S, Rottmar M (2022). The response of soft tissue cells to Ti implants is modulated by blood-implant interactions. Mater Today Bio.

[36] Ricklin D, Hajishengallis G, Yang K, Lambris JD (2010). Complement: a key system for immune surveillance and homeostasis. Nat Immunol.

[37] Leavesley DI, Kashyap AS, Croll T, Sivaramakrishnan M, Shokoohmand A, Hollier BG (2013). Vitronectin—master controller or micromanager?. IUBMB Life.

[38] Ma YJ, Garred P (2018). Pentraxins in complement activation and regulation. Front Immunol.

[39] Galván-Peña S, Carroll RG, Newman C, Hinchy EC, Palsson-McDermott E, Robinson EK (2019). Malonylation of GAPDH is an inflammatory signal in macrophages. Nat Commun.

[40] Schittek B (2012). The multiple facets of dermcidin in cell survival and host defense. J Innate Immun.

[41] Pradipta J, Mobidullah K, Subrata DK, Asru SK, Santanu G, Gausal KA (2018). Estriol inhibits dermcidin isoform-2 induced inflammatory cytokine expression via nitric oxide synthesis in human neutrophil. Curr Mol Med.

[42] Al-Mozaini MA, Tsolaki AG, Abdul-Aziz M, Abozaid SM, Al-Ahdal MN, Pathan AA (2018). Human properdin modulates macrophage: Mycobacterium bovis BCG interaction via thrombospondin repeats 4 and 5. Front Immunol.

[43] Conover CA, Clarkson JT, Bale LK (1994). Insulin-like growth factor-II enhancement of human fibroblast growth via a nonreceptor-mediated mechanism. Endocrinology.

[44] Smith YE, Toomey S, Napoletano S, Kirwan G, Schadow C, Chubb AJ (2018). Recombinant PAPP-A resistant insulin-like growth factor binding protein 4 (dBP4) inhibits angiogenesis and metastasis in a murine model of breast cancer. BMC Cancer.

[45] Renier G, Clément I, Desfaits A-C, Lambert A (1996). Direct stimulatory effect of insulin-like growth factor-I on monocyte and macrophage tumor necrosis factor-α production. Endocrinology.

[46] Du L, Lin L, Li Q, Liu K, Huang Y, Wang X (2019). IGF-2 preprograms maturing macrophages to acquire oxidative phosphorylation-dependent anti-inflammatory properties. Cell Metab.

[47] Norseen J, Hosooka T, Hammarstedt A, Yore MM, Kant S, Aryal P (2012). Retinol-binding protein 4 inhibits insulin signaling in adipocytes by inducing proinflammatory cytokines in macrophages through a c-Jun N-terminal kinase- and toll-like receptor 4-dependent and retinol-independent mechanism. Mol Cell Biol.

[48] Williams DF. Titanium for medical applications. In: Brunette, Donald M, Tengvall P, Textor M, Thomsen P, editor. Titan Med Mater Sci Surf Sci Eng Biol Responses Med Appl. Berlin: Springer; 2001. p. 12–24

[49] Bekassy Z, Lopatko Fagerström I, Bader M, Karpman D (2022). Crosstalk between the renin–angiotensin, complement and kallikrein–kinin systems in inflammation. Nat Rev Immunol.

[50] Schuijt TJ, Bakhtiari K, Daffre S, Deponte K, Wielders SJH, Marquart JA (2013). Factor XA activation of factor v is of paramount importance in initiating the coagulation system: lessons from a tick salivary protein. Circulation.

[51] Miles LA, Ny L, Wilczynska M, Shen Y, Ny T, Parmer RJ (2021). Plasminogen receptors and fibrinolysis. Int J Mol Sci.

[52] Neering SH, Adyani-Fard S, Klocke A, Rüttermann S, Flemmig TF, Beikler T (2015). Periodontitis associated with plasminogen deficiency: a case report. BMC Oral Health.

