# Supplementary material for: Roughness affects the response of human fibroblasts and macrophages to sandblasted abutments
Source: Biomed Eng Online. 2024 Jul 17;23:68. doi: 10.1186/s12938-024-01264-6 (PMC11253364; doi:10.1186/s12938-024-01264-6)
Supplement: Supplementary file 1 — Additional file 1. [file 12938_2024_1264_MOESM1_ESM.pdf]

Additional File 1. Perseus results of the comparative analysis between the proteins identified onto the MC04 and MC08 surfaces with respect to MC. Differential proteins show a p ≤ 0.05 (yellow) and a ratio higher than 1.5 in either direction. Red indicates proteins more adsorbed on sandblasted surfaces, while green marks those less adsorbed.

			MC04/MC		MC08/MC	
Uniprot code	Protein name	Unique peptides	p value	Ratio	p value	Ratio
HV313	Immunoglobulin heavy variable 3-13	1	1,8E-01	2,9	2,8E-02	4,3
KNG1	Kininogen-1	26	1,2E-05	2,4	3,0E-04	2,3
HV169	Immunoglobulin heavy variable 1-69	1	2,2E-03	2,0	2,0E-01	2,2
KLKB1	Plasma kallikrein	16	3,6E-05	2,5	2,3E-04	2,2
APOF	Apolipoprotein F	3	8,0E-02	1,3	2,5E-03	1,7
IGF2	Insulin-like growth factor II	2	8,9E-03	6,4	3,8E-01	1,7
APOL1	Apolipoprotein L1	6	2,8E-04	1,8	4,9E-04	1,6
CO6	Complement component C6	8	1,9E-04	1,8	1,7E-02	1,6
CO9	Complement component C9	12	2,7E-06	1,9	3,0E-04	1,6
FA11	Coagulation factor XI	31	4,2E-03	1,6	5,4E-03	1,6
CO8A	Complement component C8 alpha chain	8	6,0E-03	1,7	3,9E-02	1,6
HV372	Immunoglobulin heavy variable 3-72	2	8,1E-03	1,5	1,3E-04	1,5
PLMN	Plasminogen	36	3,6E-04	1,5	7,7E-04	1,5
ANT3	Antithrombin-III	23	5,8E-04	1,9	2,3E-04	1,5
ZPI	Protein Z-dependent protease inhibitor	7	3,8E-02	2,9	4,8E-01	1,5
VTNC	Vitronectin	21	7,0E-06	1,9	5,9E-04	1,5
KV401	Immunoglobulin kappa variable 4-1	4	3,2E-02	1,7	1,7E-02	1,5
APOC2	Apolipoprotein C-II	3	3,9E-03	1,5	4,7E-02	1,4
APOB	Apolipoprotein B-100	87	1,4E-02	1,8	1,1E-01	1,4
HV551	Immunoglobulin heavy variable 5-51	4	5,1E-06	1,9	6,1E-03	1,3
HV459	Immunoglobulin heavy variable 4-59	2	3,3E-03	1,5	3,2E-02	1,3
APOC3	Apolipoprotein C-III	2	1,3E-02	1,6	1,9E-01	1,2
IBP4	Insulin-like growth factor-binding protein 4	2	6,5E-05	2,3	4,0E-01	1,2
RL40	Ubiquitin-60S ribosomal protein L40	3	7,6E-03	2,7	8,7E-01	1,1
CRP	C-reactive protein	5	1,5E-03	2,0	5,8E-01	1,1
CO5	Complement C5	20	2,3E-03	2,0	8,0E-01	1,1
APOC4	Apolipoprotein C-IV	4	1,9E-03	0,6	6,5E-01	0,9
RET4	Retinol-binding protein 4	5	1,1E-02	0,6	6,1E-02	0,8
C1QA	Complement C1q subcomponent subunit A	2	4,1E-03	0,5	3,2E-02	0,7
XP32	Skin-specific protein 32	2	3,4E-02	0,5	3,7E-01	0,7
DCD	Dermcidin	5	2,6E-03	0,4	4,5E-02	0,5
PROP	Properdin	5	3,1E-01	0,8	2,8E-03	0,5
SAMP	Serum amyloid P-component	10	3,1E-06	0,5	6,4E-03	0,5
FA5	Coagulation factor V	6	4,5E-03	0,6	1,0E-01	0,4
FHR5	Complement factor H-related protein 5	5	2,4E-02	0,7	8,7E-04	0,4
HABP2	Hyaluronan-binding protein 2	16	3,8E-01	0,6	7,7E-03	0,3
DSG1	Desmoglein-1	14	1,9E-01	0,3	3,3E-02	0,2
DSC1	Desmocollin-1	6	3,1E-02	0,2	3,6E-02	0,2
G3P	Glyceraldehyde-3-phosphate dehydrogenase	5	1,1E-02	0,4	1,1E-02	0,1
